# Post-Processing Trimming of Silicon Photonic Devices Using Femtosecond Laser

**DOI:** 10.3390/nano13061031

**Published:** 2023-03-13

**Authors:** Yating Wu, Hongpeng Shang, Xiaorui Zheng, Tao Chu

**Affiliations:** 1 College of Information Science and Electronic Engineering, Zhejiang University, Hangzhou 310027, China; 2 Key Laboratory of 3D Micro/Nano Fabrication and Characterization of Zhejiang Province, School of Engineering, Westlake University, Hangzhou 310030, China

**Keywords:** integrated photonics, fabrication error compensation, femtosecond laser, cladding, micro-ring, Mach-Zehnder interferometer

## Abstract

Fabrication errors inevitably occur in device manufacturing owing to the limited processing accuracy of commercial silicon photonic processes. For silicon photonic devices, which are mostly processing-sensitive, their performances usually deteriorate significantly. This remains an unsolved issue for mass production, particularly for passive devices, because they cannot be adjusted once fixed in processes. This study presents a post-processing trimming method to compensate for fabrication errors by changing the cladding equivalent refractive indices of devices with femtosecond lasers. The experimental results show that the resonant wavelengths of micro-ring resonators can be regularly shifted within their free spectral range via tuning the illuminating area, focusing position, emitting power, and scanning speed of the trimming femtosecond laser with an acceptable loss increase. These experiments, as well as the trimming experiments in improving the phase balance of Mach-Zehnder interferometer switches, indicate that the femtosecond laser trimming method is an effective and fast method for silicon photonic devices.

## 1. Introduction

Recently, silicon photonic integrations, based on complementary metal oxide semiconductor (CMOS)-compatible processes, have shown great application prospects in optical communications, optical interconnections, optical sensing, and optical computing because of their compact size, low power consumption, and low cost [1,2,3,4,5,6,7,8,9,10,11,12,13,14]. The process nodes used by mainstream silicon photonic manufacturers are usually 130 nm or 180 nm for low-cost production, which inevitably introduces fabrication errors in the devices. A process error of approximately tens of nanometers can usually be found in commercial 130 nm silicon photonic processes [15,16,17,18,19,20]. These size variations of waveguides cause changes in the optical mode’s effective refractive indices and light propagating phases, degrading the device’s performance significantly. The unexpected fabrication error is presently a major obstruction in the mass production of silicon photonic devices, particularly for passive devices that cannot be adjusted in operations once fixed in processes. The best way to solve this issue is to design devices with a large fabrication tolerance [18,19], which projects higher requirements for design and is difficult to realize. Currently, a common method is to use the thermo-optic effect [18,21,22] to adjust the refractive index of the waveguide core, which simultaneously increases the device footprint, controlling complexity and power consumption. Therefore, a promising solution is to find a post-processing trimming method, and many efforts have been devoted to this challenge.

Existing nonvolatile post-processing trimming methods usually change the refractive index of the waveguide core or cladding through various post-processing methods, mainly including the following types of schemes: 1. Switch the waveguide core material between the crystalline and amorphous states by illuminating the waveguide with the femtosecond (Fs) laser [23,24,25,26,27], thermal annealing after germanium (Ge) implantation [28,29], and so on. The Fs laser converts the silicon waveguide from a crystalline state to an amorphous state, increasing the waveguide core refractive index. This technology adopts a single laser shot with little additional light propagation loss but lacks processing accuracy [23,24,25,26,27]. Ge implant makes the waveguide amorphous, which can recrystallize rapidly through annealing. Nevertheless, Ge implantation at wafer scale inevitably increases chip loss, and thermal annealing also increases energy consumption [28,29]. 2. Oxidize the waveguide core to reduce its height through the atomic force microscope [30], continuous-wave lasers [31], or high-intensity lasers [23,24,26], thereby reducing the effective refractive index of the optical modes, but probably introducing significant extra loss and limiting the tuning range. 3. Use polymers or other special cladding materials sensitive to visible light [32,33], ultraviolet irradiation [34], or electron beams [35]. Their disadvantages include incompatibility with CMOS processes, poor timing stability, and high-temperature sensitivity. 4. Deposit silicon nitride or other special claddings, and strictly control the trimming pattern of the cladding to adjust the waveguide light confinement [36]. This increases the process complexity and complicates limiting the deposition area. 5. Use electron beam-induced compaction upon silica cladding to introduce strains into the waveguide core [37]. This method is costly and difficult to control and repeat since the strains relax over time.

Among the methods mentioned above, Fs laser direct writing has been widely used to process photonic devices because of its maskless, three-dimensional, and precise processing [38,39,40,41,42,43,44]. It is reported that the resonant wavelength (RNWL) redshifts and blueshifts under different Fs laser processing on a silicon waveguide core without cladding [23,24,25,26,27]. However, almost all silicon photonic devices in practical applications are covered by silica claddings for protection. Researchers have also reported that the RNWL redshifts after processing the silicon waveguide core through transparent cladding by an Fs laser [25,26,27]. However, this requires further investigation, as a practical method is required for specific applications.

In this paper, we present a post-processing trimming method to compensate for fabrication errors with an Fs laser upon silica cladding, without significantly affecting the core layer. The method was experimentally verified using micro-ring resonators (MRR) and phase-biased Mach-Zehnder interferometer (MZI) switches with forward-biased PIN junctions as phase shifters [45,46]. By changing the illuminating area, focus position, emitting power, and scanning speed of the Fs laser, corresponding regular curves were obtained, which provided references for the quantitative adjustment of the MRR RNWL. Moreover, by applying the Fs laser to compensate for the phase bias in MZI-typed electro-optic switches, we improved the performance uniformity between the BAR and CROSS states, reducing the power consumption during operation. The post-processing trimming method by an Fs laser has shown a large adjustment range and fast trimming within approximately a few seconds, providing a new solution for dealing with fabrication errors, especially for large-scale integrated silicon photonic systems.

## 2. Experiments

**Figure 1:**
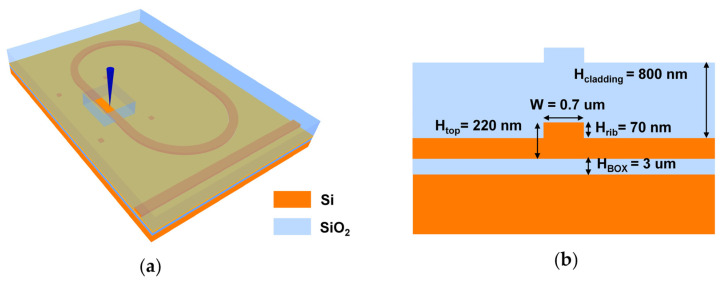
Diagrams of (**a**) Fs laser post-processing trimming upon micro-ring resonators (MRR); (**b**) the waveguide cross section.

In the experiments, the Fs laser illuminated the straight waveguides of the racetrack MRRs to investigate the trimming effects on the RNWL shift, as shown in Figure 1a. The MRRs were fabricated on a silicon-on-insulator (SOI) substrate with a 220-nm thick top silicon layer (H_top_) and a 3-μm thick buried oxide layer (H_BOX_), as shown in Figure 1b. Moreover, this device was covered with a 0.8-μm thick silica upper cladding layer (H_cladding_). The ridge waveguide was used with a width of 0.7 μm (W) and an etching depth of 70 nm (H_rib_). The gap of the directional coupler in the MRRs was 0.18 μm. The length of the straight waveguide perpendicular to the MRR bus waveguide was 30 μm, which was sufficient for Fs laser post-processing experiments.

The MRR resonant spectra were simulated with different cladding equivalent refractive indices, using Ansys Lumerical (2021 R2.5, Pennsylvania America) software and Python (3.9.12) programs. The simulation steps and settings were as follows:

1. An SOI platform with silica cladding of a 1.45 refractive index was constructed, and waveguides from the ring of the MRR were added in FDE software. An 8-μm wide and 5-μm high simulation area was symmetrically placed around the cross-section of the waveguide, in which boundary conditions of PML and background material of air were used. Moreover, an extra mesh of 0.015 μm covering the waveguide was added to improve the precision of the simulation.
2. Frequency analysis was conducted in a band ranging from 1.305 μm to 1.315 μm, and the complex refractive index of the mode TE0 as a function of wavelength was extracted.
3. The change in the cladding index was adjusted from −0.1 to 0.1, and step 2 was repeated.
4. The output power of the directional coupler in the MRR was simulated from 1.305 μm to 1.315 μm through Lumerical FDTD software at mesh 3.
5. The above results from steps 2 and 4 were substituted into the spectral formula of the MRR, and the resonant spectra with different equivalent refractive indices of cladding were obtained via Python programs.

**Figure 2:**
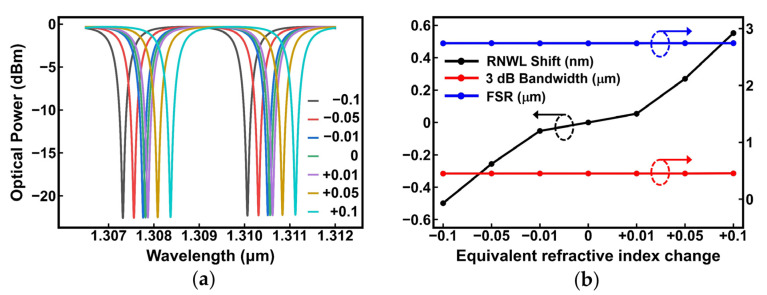
Simulations of MRRs. (**a**) Optical spectra with different cladding equivalent refractive indices; (**b**) resonant wavelength (RNWL) shift, 3 dB bandwidth, and free spectral range (FSR) vs. change in cladding equivalent refractive index.

When the equivalent refractive index of the cladding increased or decreased from 1.45, the RNWL redshifted and blueshifted, respectively, as shown in Figure 2a. The RNWL shift, 3 dB bandwidth, and free spectral range (FSR) were extracted and plotted in Figure 2b, in which the RNWL shift was positively correlated to the equivalent refractive index change in the cladding. Furthermore, 3 dB bandwidth and FSR were independent of the index change, indicating no extra loss. Therefore, adjusting the equivalent refractive index of the cladding might be a promising method for post-processing trimming because it is effective and does not cause additional losses.

**Figure 3:**
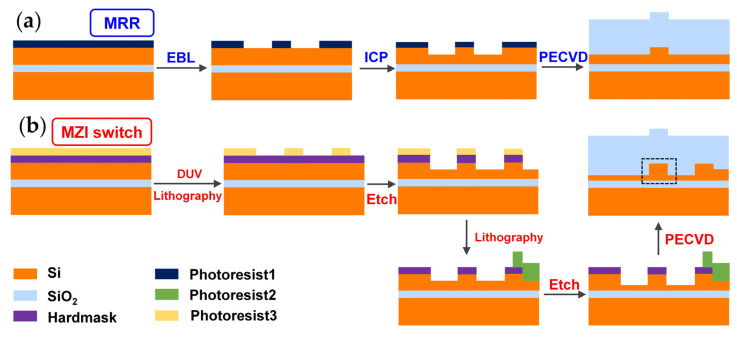
Fabrication processes of waveguides in (**a**) MRRs; (**b**) Mach-Zehnder interferometer (MZI) switches.

**Figure 4:**
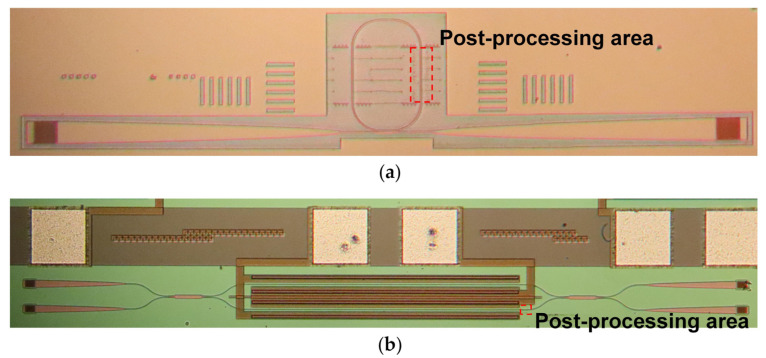
Microscopy views after post-processing trimming: (**a**) MRR; (**b**) MZI switch.

The detailed fabrication process is shown in Figure 3a. The pattern was defined by electron-beam lithography (EBL) and then transferred to the top silicon layer by inductively coupled plasma (ICP) dry etching. An 80-nm-thick cladding layer was deposited on the waveguides using plasma-enhanced chemical vapor deposition (PECVD). Processing marks were added near the straight waveguides as trimming references. More than 100 MRRs with the same parameter were fabricated, as shown in Figure 4a. We tested the devices before and after trimming for comparison. The light source was launched from a tunable laser (Santec TSL-550), followed by a polarization controller. An optical power meter (Yokogawa, AQ2211) was used to receive the output power from the chip. The wavelength sweep was performed from 1280 nm to 1300 nm in steps of 0.005 nm under control programs.

For the MRR experiments, we changed the focus position, scanning speed, illuminating power of the Fs laser, and processing area to investigate the trimming effects. The PHAROS2-1mJ-SP-typed Fs laser we used was an all-in-one integrated Fs laser with adjustable parameters, including pulse width (<190 fs), repetition rate (single pulse to 1 MHz), single pulse energy (up to 1 mJ), and average emitting power (up to 10 W). The laser’s wavelength was fixed at 405 nm. The MRRs on the SOI chip were irradiated by a focused Fs laser beam through a microscope with a 20× microscope objective.

In addition, MZI switches, fabricated by the Singapore Advanced Micro Foundry Company, were also trimmed by the Fs laser, as shown in Figure 4b. The electro-optic switches had 3-μm-thick top and bottom claddings, a 220-nm-thick top silicon layer, and 500-nm-wide ridge waveguides with a 130-nm-etching depth. Only the fabrication process of the passive waveguide was shown in Figure 3b.

Table 1 illustrates the differences between MRRs and MZI switches. Deep ultraviolet (DUV) lithography and EBL were adopted, respectively, and the thicknesses of the cladding layer were 0.8 μm and 3 μm, respectively. The depths of the waveguide core were both 220 nm, but the etching depths were 70 nm and 130 nm, respectively. MZI switches worked in the C band, and MRRs worked in the O band, both of which were coupled by the grating couplers.

## 3. Results and Discussion

### 3.1. Working Mechanism

According to previous studies, it was discovered that the increasing melting and vaporization of the surface silicon led to the removal of the bulging silica layer [47,48,49,50,51,52], which was suspected to result in a decrease in the equivalent refractive index of the cladding and the effective refractive index of the waveguide modes, and then the RNWL blueshift occurred. Partial removal of the cladding after the strong post-processing trimming was observed during the experiments, consistent with the previous reports. However, not all post-processing areas were observed to change in geometry, and the working mechanism was considered complex when the laser was focused in the cladding. It was possible that the change in cladding geometry was too microscopic or that the direct modification of optical properties occurred rather than the change in morphology. Moreover, it was difficult to observe the change in the morphology of the waveguide after trimming because of the deposition of cladding.

### 3.2. Fabrication Tolerance

#### 3.2.1. MRRs

**Figure 5:**
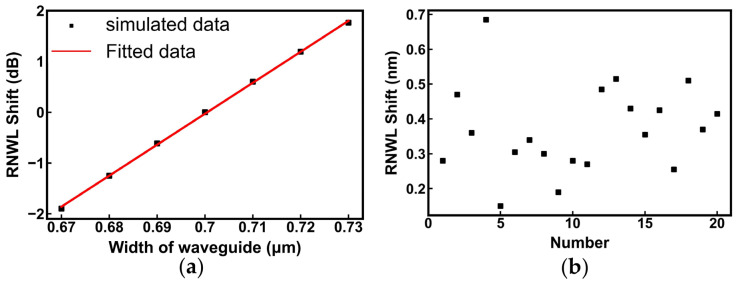
(**a**) Simulation for RNWL Shift vs. the width of the waveguide; (**b**) measured RNWL Shift of 20 MRRs.

For MRRs, the RNWL shift was determined by the optical path of a loop, whose most relevant parameter was the width of the waveguide in the ring. Assuming that the coupling coefficient of the directional coupler in MRRs was fixed, the waveguide width of the ring was changed from 0.67 μm to 0.73 μm, and a cosimulation through Lumerical FDE software and Python programs was conducted to obtain the optical spectra of MRRs under different waveguide widths, as shown in Figure 5a. The change in the waveguide width of approximately 16.3 nm resulted in an RNWL shift of 1 nm. Considering a 3 dB bandwidth of 0.45 nm as the tolerable RNWL shift, the process tolerance of the MRR is approximately ±3.7 nm.

Compared with the target RNWL, the RNWL shift of fabricated MRRs ranged from +0.2 nm to +0.7 nm in Figure 5b, indicating that the actual process error was at least 11 nm and exceeded the above process tolerance. According to the relationship curves between the parameters of the laser and the RNWL shift, and the exact shift from the target value, the specific setting of parameters can be calculated for the compensation.

#### 3.2.2. MZI Switches

**Figure 6:**
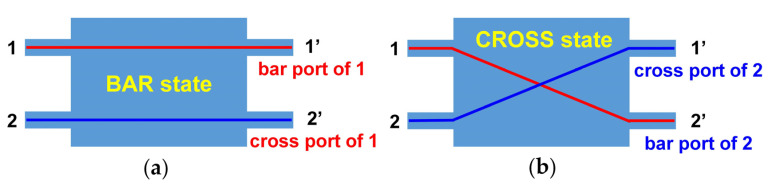
Two states of the MZI switch unit. (**a**) BAR state; (**b**) CROSS state.

The MZI switch unit consists of two input ports and two output ports. As shown in Figure 6, when light is input from port 1 (2), port 1′ (2′) is named the bar port, while port 2′ (1′) is named the cross port corresponding to the input port. The BAR state is defined as the state where the optical power (OP) of the bar port is maximum, as shown in Figure 6a below, while the CROSS state is defined as the state where the optical power of the cross port is maximum, as shown in Figure 6b.

**Figure 7:**
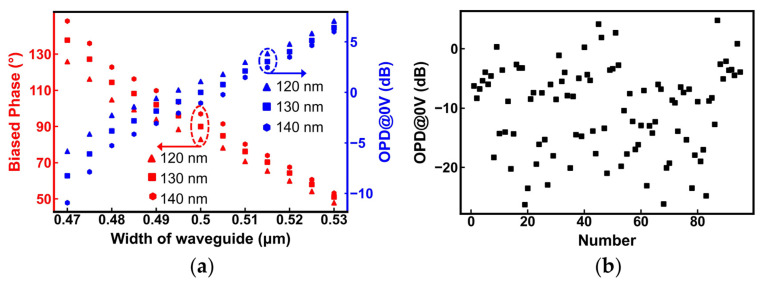
(**a**) Simulation for biased phase and optical power difference at 0 V (OPD@0 V) vs. width of waveguide under etching depths of 120 nm, 130 nm, and 140 nm; (**b**) measured OPD@0 V of approximately 90 MZI switches.

Although the implemented 90° phase bias effectively improves the balance of the switch [46], it is still greatly affected by process errors, causing an uneven extinction ratio (ER) and power consumption of the BAR and CROSS states, even for the same batch of MZI switches fabricated. The waveguide width of the phase bias in the MZI switch was swept under different etching depths to get the curves in Figure 7a. When the waveguide width deviated from the ideal width of 500 nm with an etching depth of 130 nm, the biased phase detached linearly from the perfect 90°, and the optical power difference (OPD) at the voltage of 0 V (OPD@0 V) also changed linearly. The changes were more drastic at larger etch depths. Here, acceptable OPD@0 V was limited to ±5 dB, so the process tolerance of the waveguide width was approximately ±20 nm to sustain the two states. The process errors of the dies in different positions of the wafer were approximately ±25 nm according to measurements, which were beyond its process tolerance, and the measured OPD@0 V ranged from 5 dB to −28 dB for 90 MZI switches, as shown in Figure 7b. Therefore, the phase should be corrected by post-processing trimming in practical applications.

### 3.3. MRRs after Post-Processing Trimming

#### 3.3.1. RNWL Shift vs. Focus Position

In these experiments, we changed the Fs laser focus position from −3.4 to 3.4 μm while keeping the laser power, laser scanning speed, and processing area constant, which were 0.22 mW, 100 μm/s, and 30 μm × 8 μm (length × width of trimming area), respectively. We defined a specific height as the critical relative position, at which the color of the processing area appeared faintly white, and the Fs laser began to work preliminarily. Its corresponding focus position was 0 μm. The relative focus position (RFP) was changed by adjusting the height of the displacement stage. When the RFP was positive, the laser focus was close to the waveguide, and the laser beam moved away from the waveguide at a negative RFP.

**Figure 8:**
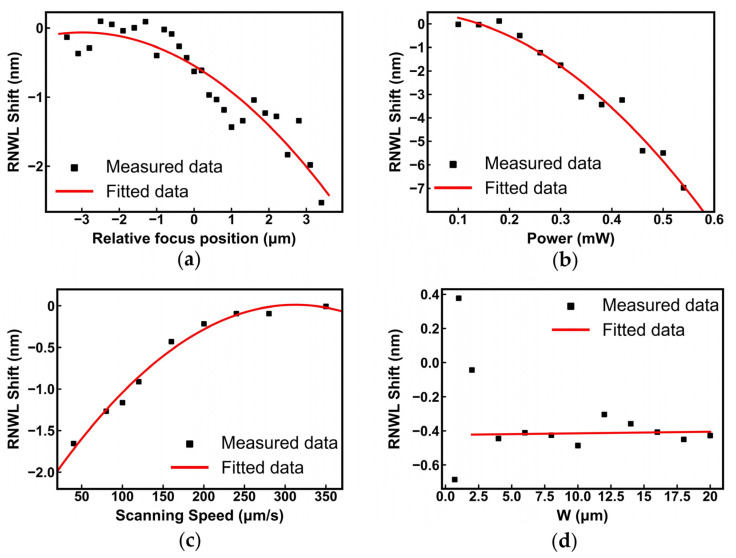
Measurements after post-processing trimming. RNWL shift vs. (**a**) focus position; (**b**) power; (**c**) scanning speed of the Fs laser; and (**d**) width of the processing area.

We measured the spectra of MRRs before and after post-processing trimming and extracted the RNWL shift curve in Figure 8a. The regularity between the RNWL shift and the focus position shows a positive correlation overall. When the focus position changes gradually from −3.4 to 3.4 μm with a 0.2 μm step, the laser focus position moves from the air at the top cladding layer to the upper surface of the silicon layer.

When the focus position changes from −3.6 μm to −1 μm, almost no RNWL shift is triggered by the Fs laser because the distance between the focus position and the upper surface of the cladding is at least 0.2 μm if we consider the critical focus position to be accurate, and the energy in the SOI chip is limited. When the focus position moves from −1 μm to 1 μm, the RNWL shift is linear with the movements of the focus position, and its slope is probably 0.78 nm/μm. That is, because the focus position gradually moves closer to the cladding and waveguide core layer, the energy affecting the surface silicon and cladding increases, and the modification to the cladding increases gradually, resulting in larger phase compensation. When the RFP is larger than 1 μm, it deviates from the above regularity. In this range, the energy of the femtosecond laser acting on the silicon layer increases further, and the working mechanism is suspected to change, mainly manifesting in the thickness thinning of the silicon waveguide [23,24,26], after the silica cladding is completely broken.

#### 3.3.2. RNWL Shift vs. Laser Power

In these experiments, we controlled the laser focusing on the cladding at approximately −0.4 μm and changed the laser power from 0.1 mW to 0.54 mW while keeping the scanning speed and processing area unchanged, which were 100 μm/s and 30 μm × 8 μm, respectively. Figure 8b shows that the relationship positively correlates with the Fs laser power, similar to a quadratic fitting curve. When the laser power is less than 0.2 mW, there is almost no RNWL shift, because the laser power is too small, and the cladding equivalent refractive index does not undergo modification. However, when the power is greater than 0.2 mW, a significant blueshift begins to appear with a slope of at least 0.63 nm/0.04 mW, which is mainly influenced by the expanded physical modifications of the silica cladding with increasing power. Several points deviate slightly from the fitted curve, possibly owing to the unstable laser power output.

It is worth noting that the RNWL shift is larger than that of three FSRs with a laser power of 0.57 mW, and there may be a larger adjustment range when increasing the power to the limit value. Therefore, this post-processing method has a broad adjustment range.

#### 3.3.3. RNWL Shift vs. Scanning Speed of the Fs Laser

In these experiments, we changed the scanning speed from 40 μm/s to 350 μm/s and kept the focus position in the cladding at approximately −0.4 μm with a laser power of 0.3 mW and a processing area of 30 μm × 8 μm unchanged.

Figure 8c shows that the RNWL shift increases with a slower scanning speed. Since the Fs laser stays longer in the dwelling area at slow scanning speeds, the modification of silica and change in the cladding equivalent refractive index are enlarged, and the RNWL shift increases with a stronger trimming effect.

#### 3.3.4. RNWL Shift vs. Width of the Trimming Area

We changed the width of the processing area perpendicular to the straight waveguide from 0.7 μm to 20 μm, while the focus position in the cladding of approximately −0.4 μm, laser power of 0.22 mW, scanning speed of 100 μm/s, and length of the processing area of 30 μm were kept unchanged in these experiments.

Figure 8d shows that the RNWL shift is stable at approximately 0.4 nm when the width of the processing area is greater than 2 μm. When the trimming width is 12 μm or 14 μm, the RNWL shift is less than 0.1 nm from the stable value, which may be due to the instability of the output power, and after correcting the power, the subsequent offset returns to the stable value. This can be improved by using more accurate power monitoring devices.

However, when selecting a processing width not greater than 2 μm, the RNWL shift deviates from the stable value for two main reasons. First, the limited process accuracy leads to a large deviation in the processing area, which has a significant effect on processing a narrow area. Second, the Fs laser beam stays at the endpoint of the processing line longer than the middle of the line under our control programs and results in greater effects on the endpoints, probably exacerbating the modification of the cladding and changing the thickness of the ridge or slab layer of the waveguide randomly, especially when endpoints are on or adjacent to the waveguide. These complex reasons may lead to a large blueshift or a redshift when the trimming width is smaller than 3 μm. Generally, when the width of the processing area is larger than 2 μm, the position deviation caused by the processing accuracy is far from the waveguides, and the cladding environment around the waveguide is very stable. Therefore, in subsequent processing, it is relatively stable to take a processing width of at least 4 μm.

#### 3.3.5. RNWL Shift vs. Length of the Trimming Area

In these experiments, we changed the length of the processing area from 6 μm to 60 μm with a 6-μm step, while the focus position in the cladding of approximately −0.4 μm, laser power of 0.3 mW, scanning speed of 100 μm/s, and width of the processing area of 8 μm were unchanged.

**Figure 9:**
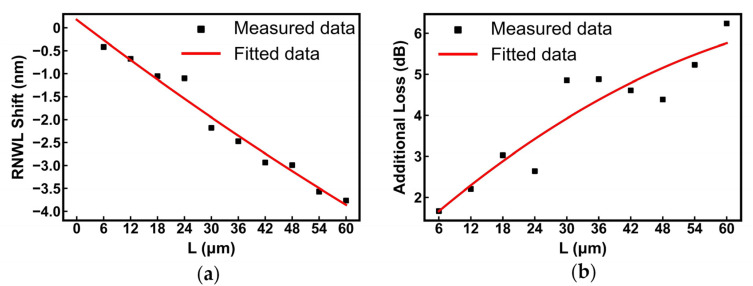
The effect of changing the processing length on the performance indicators of MRR. (**a**) RNWL shift vs. length; (**b**) additional loss vs. length.

Figure 9a illustrates that the RNWL shift becomes larger with the longer length of the trimming area owing to the further modified cladding, presenting a positively linear relationship. The required RNWL shift is usually less than that of the FSR, and a trimming length of less than 36 μm is sufficient, as shown in Figure 9a. The data point with a length of 24 μm is slightly offset, possibly owing to the unstable laser power output.

In our experiments, the processing speed of the laser was often set to 100 μm/s. Considering the longer residence time at the end of the processing line, it only took approximately 4 s to process a 30 μm × 8 μm area, and the RNWL shift of approximately one FSR was achieved, as shown in Figure 9a. Referring to [53], we also obtain additional insertion loss of the MRR loop resulting from the trimming upon length, as shown in Figure 9b. The curve is positively correlated overall, and the slope of the loss is less than 0.1 dB/μm because the Fs laser might increase the roughness of the top surface and sidewall of the silicon waveguide and cause losses.

#### 3.3.6. Synergistic Effect

Under the control variable experiments, the sweep of relative focus position, laser power, and trimming length has induced a phase adjustment that is larger than an FSR of MRRs. In this case, it is sufficient and easy to adjust a single parameter, especially when a linear relationship curve exists, such as the RNWL shift vs. the trimming length in MRRs. However, when the change in a single parameter cannot cover the expected adjustment range, it is necessary to investigate multiparameter collaborative experiments.

We believe that the trend of a single parameter sweep can provide a reference for qualitative multiparameter work. Take MRRs, for example. RFP, the power, and the trimming length positively correlated with the RNWL, while scanning speed negatively correlated. It is considered that with a slower sweep speed, and a larger RFP, power, and length, there is a positive correlation relationship between the RNWL shift and the changed parameters, whose slope is even greater than the sum of that from all the changed parameters. More experiments are required for precise and quantitative analysis.

### 3.4. Switches after Post-Processing Trimming

**Figure 10:**
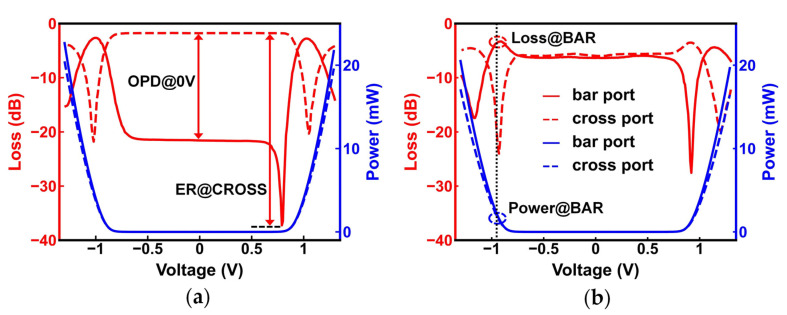
V−L curves and V−P curves of an MZI switch (**a**) before trimming; (**b**) after trimming.

We also measured the performance of the MZI switches before and after trimming, as shown in Figure 10. The red curves indicate the relationship between the loss of switch and voltage (V–L), and the blue curves indicate the relationship between power consumption and voltage (V–P). The solid curves and the dashed curves indicate the performance of the bar and cross ports, respectively. We extracted various performance indicators from Figure 10 and listed them in Table 2. After trimming, the performance indicators of the MZI switch in the BAR and CROSS states, such as ER, loss, drive voltage, and power consumption, became more balanced.

The CROSS state is more easily achieved than the BAR state for single-driven 2 × 2 switches without phase-bias designs [46]. In the push–pull drive state, a perfect 90° phase bias compensates for the phase difference of the first 3 dB beam splitter, and the OPD between the two output ports of the switch should be equal at 0 V voltage. However, the V–L curves in Figure 10a show that the measured OPD of a push–pull driven MZI switch is approximately −19.8 dB with no voltage, indicating that the phase bias is an off-perfect value of 90°. After applying voltage, the CROSS state is achieved with a voltage of 0.79 V, smaller than a voltage of −1.02 V in the BAR state, indicating that the actual phase bias is insufficient. Similarly, the drive voltage and ER of the BAR state and the CROSS state before trimming also have significant differences due to insufficient phase bias, which are 0.23 V and 14.5 dB, respectively.

To compensate for the phase bias, we used the Fs laser to process an area of 9 μm × 8 μm in the MZI lower arm, as shown in Figure 4b. In these experiments, we set the Fs laser focus position to −0.5 μm, the laser power to 0.6 mW, and the laser scanning speed to 100 μm/s, respectively. The red V−L curves in Figure 10b illustrate that the OPD@0 V is less than 0.5 dB after trimming. Moreover, the balance of performance is significantly improved, and the two-state differences in drive voltage and ER are 0.01 V and 3.15 dB, respectively. The difference in the power consumption of this switch in the two states is also reduced from 4.54 mW to 0.1 mW, as shown by the V–P curves before and after trimming in Figure 10. Unfortunately, there are 0.64 dB and 1.68 dB losses at the BAR and CROSS states, respectively, which are probably due to the unexpected effect on the waveguide.

## 4. Conclusions

This study successfully demonstrated a method to compensate for fabrication errors of integrated silicon photonic devices by changing the cladding equivalent refractive index with post-processing trimming of the Fs laser. Our results show that:

- Trimming on silica cladding by the Fs laser with a large adjustment range and fast trimming, brings the regular blueshift of the MRR RNWL with different parameter changes and significantly improves the performance balance for MZI switches after compensating for phase-biased devices.
- When the focus position is close to the cladding and silicon waveguide core, the RNWL blueshift gradually increases, and the relationship is significantly positively correlated.
- The RNWL blueshift increases with increasing laser power, showing a square correlation.
- The RNWL blueshift increases with reduced scanning speeds.
- When the width of the processing area is greater than 4 μm, the RNWL shift is stable for waveguides with a 0.7 μm width.
- The RNWL shift has a linear relationship with the trimming area length, which is a stable and promising method for post-processing trimming.

Therefore, for platforms with the cladding of silica such as SOI and silicon nitride on the insulator [49,54], this method can be considered to compensate for phase-sensitive devices or systems, such as optical computing chips for the photonic neural network [13], optical modulators [55], wavelength division (de) multiplexers [14], and beam splitter devices like directional couplers [56]. Unfortunately, the trimming accuracy of laser trimming in batches has not yet been tested, because deviations in the relative focus position and the output power exist in the experiments, which must be improved in subsequent experiments. More flexible control methods, including improving the precision of control programs and the dimension of Fs laser trimming, need to be explored. Furthermore, the influence of the increase in core roughness on device loss needs to be investigated and controlled.

## Figures and Tables

**Table 1:** Differences between MRRs and MZI switches.

Device	Depth of Etching (nm)	Thickness of Cladding (μm)	Pattern Fabrication	Band (nm)
MRR	70	0.8	Electron-beam lithography (EBL)	O Band (1310)
MZI switch	130	3	Deep ultraviolet (DUV) lithography	C Band (1550)

**Table 2:** Performance indicators extracted from measured curves before and after trimming.

	OP (dBm)	State	Voltage (V)	Extinction Ratio (ER) (dB)	Power (mW)	Loss (dB)
Before trimming	−21.53 (bar)	BAR	−1.02	20.05	4.49	−2.72
	4−1.74 (cross)	CROSS	0.79	34.59	0.05	−1.89
After trimming	−6.39 (bar)	BAR	−0.93	21.08	1.45	−3.36
	−5.9 (cross)	CROSS	0.92	24.24	1.32	−3.57

## Data Availability

The data presented in this study are available on request from the corresponding author. The data are not publicly available due to privacy.
